# Transcendendo: A Cohort Study of HIV-Infected and Uninfected Transgender Women in Rio de Janeiro, Brazil

**DOI:** 10.1089/trgh.2018.0063

**Published:** 2019-04-05

**Authors:** Ana Cristina Garcia Ferreira, Lara Esteves Coelho, Emilia Moreira Jalil, Paula Mendes Luz, Ruth K. Friedman, Maria Regina C. Guimarães, Rodrigo C. Moreira, Leonardo F. Eksterman, Sandra Wagner Cardoso, Cristiane V. Castro, Monica Derrico, Ronaldo I. Moreira, Biancka Fernandes, Laylla Monteiro, Luciana Kamel, Antonio G. Pacheco, Valdilea G. Veloso, Beatriz Grinsztejn

**Affiliations:** ^1^ Evandro Chagas National Institute of Infectious Diseases, Oswaldo Cruz Foundation, Rio de Janeiro, Brazil.; ^2^ Scientific Computing Program, Oswaldo Cruz Foundation, Rio de Janeiro, Brazil.

**Keywords:** cohort, HIV, transgender health, transgender person, transgender woman

## Abstract

**Purpose:** Worldwide, the burden of adverse health conditions is substantial among travestis and transgender women (trans women). Transcendendo, the first trans-specific cohort in a low- or middle-income country, is an open cohort established in August 2015 to longitudinally evaluate the health aspects of trans women aged ≥18 years in Rio de Janeiro, Brazil.

**Methods:** Study visits occur on an annual basis. Data on sociodemographics, behavioral, gender transition, affirmation procedures, hormone use, discrimination, violence, clinical and mental health, HIV prevention, and care (for those HIV-infected) are collected. Physical examination, anthropometric measurements, and laboratory tests are performed.

**Results:** As of July 2017, 322 trans women were enrolled in the cohort with a median age of 31.5 years (interquartile range 25.7–39.5), of whom 174 (54%) were HIV-infected. The Transcendendo baseline information reinforces the scenario of marginalization and deprivation surrounding trans women. Most participants had low income (62.0% were living with below US$ 10.00/day), showed a very high engagement in sex work (78.6%), and reported increased occurrence of sexual (46.3%) and physical (54.0%) violence. Pre-exposure peophylaxis (PReP) was used by 18.8% of the HIV-uninfected trans women, only through research participation. Positive screening for depression (57.8%) and problematic use of tobacco (56.6%), cannabis (28.9%), cocaine (23.8%), and alcohol (21.5%) were high. Almost all participants (94.8%) reported hormone use at some point, mostly without medical supervision (78.7%).

**Conclusion:** Our results describe a context of exclusion experienced by trans women, exposing vulnerabilities of this population in a middle-income country, with poor access to trans-specific care, HIV prevention and care, and mental health care. Addressing transgender experiences and needs can help the development of strategies to diminish stigma, improve health care environment, guide future research on trans morbidities, substance use, and trans-specific interventions to support health-related recommendations. Ultimately, it contributes to close the gaps concerning transgender health and reinforces that trans care cannot be disentangled from the social environment that surrounds trans women.

## Introduction

Worldwide, the burden of adverse health conditions is substantial among transgender women and travestis (trans women)—people who were assigned as male at birth but identify themselves as females.^1–4^ Notwithstanding, they are an underserved group, with several gaps in knowledge regarding their health.^2^ Although there is no definitive definition, the term travesti has historical and political significance in Brazil and is mostly used to refer to people of the feminine spectrum that, in general, do not wish to undergo reassignment.^5^

Estimates showed that the transgender population size is small, ranging from 0.5% to 0.9% of the overall population^3^ but, nevertheless, has a disproportionate HIV risk.^1^ A systematic review, using data from 2000 to 2011, found that HIV prevalence reached 18% in this population, with a 50-fold increased odds of infection compared with other adults in the reproductive age.^6^ Some of the highest HIV prevalence rates occur in Latin America, where trans women live in the context of profound social exclusion and is a population most vulnerable to HIV.^7^ This vulnerability is a result of the complex interactions of risks at the individual level (condomless anal sex, substance use, sex work), interpersonal risks (high-risk partner pool), and structural factors (social exclusion, violence, discrimination, unemployment).^8,9^ Recent data showed a high HIV prevalence in this population in 12 Brazilian cities.^10^ One study recently reported 31.2% HIV prevalence among trans women in Rio de Janeiro, Brazil.^11^

Although trans women are a key population for HIV infection, there are very few tailored prevention and treatment programmes and interventions specifically designed for this population. Historically, trans women were inappropriately grouped with men who have sex with men, which not only hindered the attainment of trans-specific data but also limited their visibility in research and surveillance studies.^2,12^ Surveillance systems do not usually identify transgender respondents, and scientific data are still scarce, with a dearth of longitudinal data.^2^ To fill this gap, we established the Oswaldo Cruz Foundation (Fiocruz) Transgender Health Clinical Cohort (Transcendendo) with the primary aim of studying the health outcomes in trans women living in Rio de Janeiro, Brazil. We here describe cohort procedures in addition to trans women baseline characteristics.

## Methods

### Ethic statement

The study was reviewed and approved by the Evandro Chagas National Institute of Infectious Diseases ethics review board at Fiocruz. All information was de-identified before analysis. All participants signed an informed consent form before study procedures. The files have highly restricted access by any personnel.

### Study population

Transcendendo is a prospective, open, clinic-based cohort, established in August 2015, to longitudinally evaluate health outcomes among trans women. Inclusion criteria are: ≥18 years of age and self-reported gender identity different from male sex assigned at birth. We present the baseline data of participants enrolled from August 2015 to July 2017.

**FIG. 1.**
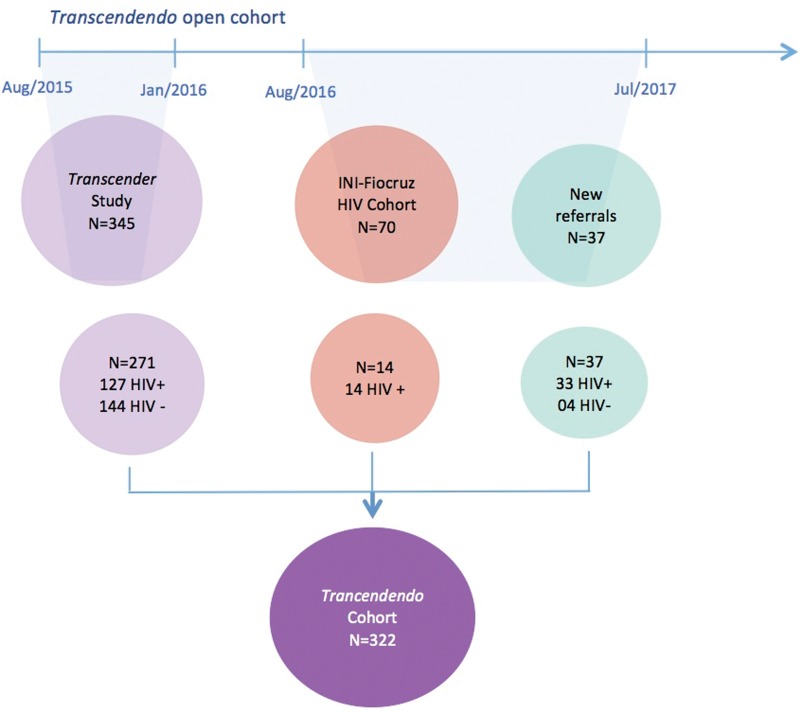
Transcendendo participant flowchart, Rio de Janeiro, Brazil, 2015–2017.

Study participants were derived from three sources, as shown in Figure 1: (1) referral from Transcender, a respondent-driven sampling (RDS) study conducted at our clinic in Fiocruz,^11^ (2) trans women who reached the site seeking participation in other studies or HIV prevention or care, and (3) from the Fiocruz HIV Clinical Cohort, described elsewhere.^13^

Trans women are a vulnerable population hardly accessible by classic sampling frame techniques for multiple reasons.^14^ A useful nonprobabilistic methodology for these populations is RDS, which is a chain referral method of recruitment. We employed RDS to recruit 345 trans women to Transcender from August 2015 to January 2016, 271 (78.5%) of whom were enrolled in Transcendendo. Every participant from Transcender had an initial visit scheduled for Transcendendo, but 74 (21.5%) did not return to the appointment (21 were not reachable with the contact information provided, 3 were no longer living in Rio de Janeiro, 14 declined to participate, and 36 were rescheduled for a second appointment but never returned).

After Transcender's closure, inclusion into our cohort was modified to any trans woman who spontaneously sought medical HIV care or prevention at our clinic. During the period from February 2016 to July 2017, 37 trans women reached our services and were enrolled in the cohort. Additionally, during this period, we reviewed the electronic records of patients from the Fiocruz HIV Clinical Cohort to identify potential candidates, which we defined as participants who were assigned as male at birth and had any mention of the term “trans” or social names in their files. This review process identified 70 trans women: as of July 2016, 12 are deceased, 15 were lost to follow-up, and 34 were potential participants. We tried to invite these 34 participants to join the cohort; 14 got enrolled as of July 2017. For the remaining 20 participants, although we tried to contact them, we hardly had any success.

To improve trans women engagement and retention, our setting presented itself as a welcoming, gender-affirming environment by using preferred names and pronouns; respecting gender identities, diversity, and expressions; providing safe, gender-neutral toilets; publicizing trans-affirming posters; offering continuous staff training on gender issues; and enabling friendly interactions between trans women and site staff. Finally, we established partnerships to facilitate social demands, such as adjustment of names in the documents, legal assistance, and providing access to potential interventions that could reduce stigma and socioeconomic disparities.

### Study procedures

#### Measures

Study visits occur on an annual basis (Table 1). Each of these visits comprises face-to-face interviews using structured questionnaires, performed by trained professionals (∼90 min), to collect detailed data on sociodemographics, sexual behavior, gender affirmation procedures, hormone and antiretroviral use (for HIV prevention or treatment), discrimination and violence, alcohol, tobacco, and drug use, physical and mental health, history of sexually transmitted infections (STIs), HIV testing history, HIV prevention, and HIV care information (for those HIV-infected).

**Table 1.** Study Procedures: Transcendendo Cohort

Study procedures	Entry evaluation	Postentry annual evaluation
Consenting	X	
Face-to-face interviews		
Sociodemographic information	X	X
Gender transition	X	
Gender affirmation procedures	X	X
Sexual behavior^a^	X	X
Discrimination^b^ and violence^c^	X	X
History of sexually transmitted infections	X	X
HIV testing history	X	X
HIV prevention knowledge and risk perception	X	X
Substance use	X	X
ASSIST questionnaire	X	
Depression screening CES-10	X	
Clinical evaluation		
Physical, mental, and medical evaluation	X	X
Hormone use	X	X
HIV care information^d^	X	X
Antiretroviral use (prevention or treatment)	X	X
Medication history	X	X
Anthropometric measurements	X	X
Laboratory testing		
Metabolic panel	X	X
Serum hormone levels	X	
Hematology and chemistry tests	X	X
STI testing	X	X
Counseling and HIV testing^e^	X	X
HIV drug resistance tests	X	
CD4^+^/CD8^+^ count and plasma HIV RNA^d^	X	X
Stored serum and plasma for future assessments	X	X

^a^ Sexual behavior was assessed through questions regarding age of sexual debut, sexual orientation, sex work, practices of receptive and insertive anal sex, vaginal sex, use of condoms, and number of partners.

^b^ Discrimination was assessed by the participant's perception of being discriminated against in the following settings: at home, school, work, health care, and on the street.

^c^ Violence was assessed with questions on sexual and physical violence ever in life and in the past year.

^d^ For HIV-infected participants.

^e^ For HIV-uninfected participants.

Procedures during visits also included clinical evaluation, as well as morbidity and medication history. We used self-reported information (prior medical diagnosis or use of specific treatment) or current results/measurements to define hypertension, diabetes, and dyslipidemia, as follows: (1) hypertension: average diastolic blood pressure ≥90 mmHg or average systolic blood pressure ≥140 mmHg,^15^ (2) diabetes: plasma nonfasting glucose ≥200 mg/dL or glycosylated hemoglobin ≥6.5%,^16^ (3) dyslipidemia: non-HDL-cholesterol ≥160 mg/dL.^17^ We considered overweight and obesity as body mass index between 25 and 29.9 kg/m^2^ and ≥30 kg/m^2^, respectively.

At baseline visit, we performed some additional procedures. We assessed information on gender perception, gender transition, and engagement in sex work. Additionally, participants responded to two validated instruments to assess depression and substance use.^18^ We screened depression using the 10-item Center for Epidemiologic Studies Depression Scale (CES-D)^19–21^ and defined it with a score of ≥10. Problematic substance use was defined when the Alcohol, Smoking, and Substance Involvement Screening Test (ASSIST) scored moderate or high risk of experiencing health problems and demanding intervention.^22^

For HIV-infected trans women, clinical information included date of first HIV-positive test, all antiretroviral therapy (ART) used, as well as other concomitant medications, history of non-AIDS-related comorbidities, and AIDS-defining illnesses, classified according to the CDC.^23^

#### Laboratory procedures

Procedures during visits also comprised laboratory testing, as described in Table 1, including STI testing (syphilis, rectal *Chlamydia trachomatis* and *Neisseria gonorrhea*, hepatitis B and C), and sample storage at all visits for future assessments. For HIV-infected trans women, we measured CD4^+^ and CD8^+^ cells and plasma viral load (VL) during every visit and performed HIV drug resistance test at baseline. For HIV-uninfected trans women who reported condomless anal intercourse in the past 30 days and have a negative HIV rapid test, we also offered pooled HIV RNA testing to identify acute HIV infection.

#### Additional data collection

We also systematically collected data on hospital admissions (causes and length of stay) and causes of death. For all deaths that occurred, we used a uniform coding system, adapted from the CoDe (“Coding of Death in HIV”) method,^24^ to revise near-death patient information either for the HIV-infected or uninfected participants. The method includes detailed data collection on the causes of death and contributing factors and a centralized review process of the collected data. Information regarding vital status was regularly checked using the patients' medical charts, via active contact with individuals and family members, and by linkage with the Rio de Janeiro state mortality database, using a previously validated algorithm.^25^

### Study definitions

#### Sociodemographic characteristics

We categorized self-reported gender identity as “woman,” “transgender woman,” “travesti,” and “other definitions.” “Travesti” is a term used in Brazil that mostly refers to individuals assigned as male at birth and have feminine gender expression, but do not identify as women, and generally do not wish to undergo feminizing vaginoplasty.^5^ Educational level included four categories, based on the number of years of formal education. Per-person daily income followed the classification of poverty.^26^ Participants born outside Rio de Janeiro State were classified as internal migrants.

#### STI diagnosis

We considered active syphilis as a positive treponemal test (rapid immunochromatographic syphilis point-of-care or microhemagglutination assay for *Treponema pallidum*) plus a positive nontreponemal test Venereal Disease Research Laboratory (VDRL) with titers of at least 1/8 at baseline. Active hepatitis B was defined as a positive HB antigen and a negative anti-HB antigen. HCV prevalence was defined as positive anti-HCV.

#### Trans-specific characteristics

Feminizing procedures included gender-affirming surgeries (breast augmentation, facial feminization, tracheal shave) and sex reassignment surgeries (SRS) (penectomy, orchiectomy, vaginoplasty). We classified the use of feminizing hormonal therapy (FHT) as “never,” “current,” or “former,” according to the report within the last 30 days. As trans women might be using more than one regimen; we inquired on every hormonal medication and classified them as “ethinylestradiol,” “intramuscular estradiol plus progestogen,” “estradiol plus antiandrogen,” or “other.” We considered “estradiol plus antiandrogen” as an adequate FHT, according to current guidelines^27–30^ and the use of ethinylestradiol or intramuscular progestagen as inadequate, as these substances are not recommended by international guidelines.^27–30^ We considered serum testosterone and estradiol levels, respectively, of <55 ng/dL and between 40 and 199 pg/mL as the target levels for FHT.^31^

### Statistical analysis

Descriptive characteristics of cohort participants are given as counts, percentages, medians, and interquartile ranges (IQR). We compared baseline characteristics of participants by HIV status using chi-squared test for categorical variables and Kruskal–Wallis test for continuous variables. In addition, we compared clinical and laboratory data for HIV-infected trans women by age strata.

## Results

As described in Table 2, 322 trans women were enrolled from August 2015 to July 2017, of whom 174 (54.0%) were HIV-infected. Median age was 31.5 years (IQR 25.7–39.5). The majority of them self-declared as nonwhite. Overall, participants reported low schooling and income, with 62.0% of them living with below US$ 10.00 per day. Sexual debut occurred before the age of 10 years for 70.2% of the participants. We observed a high prevalence of physical (*n*=174; 54.0%) and sexual (*n*=149; 46.3%) violence. Out of the 149 participants who reported a history of sexual violence, 95 (63.8%) informed that it occurred the first time before the age of 18 years, and for 58 (38.9%), it occurred before the age of 14 years. The frequency of past engagement in sex work was high (78.6%). Overall, 186 (57.6%) of trans women had a positive treponemal test, and 27.0% had active syphilis at baseline. The prevalence of active rectal *C. trachomatis* and *N. gonorrhea* was 12% and 6%, respectively, and few had active hepatitis B and C. From the 148 HIV-uninfected participants, only 18 (12.2%) were on HIV pre-exposure prophylaxis (PrEP), all of them via participation in a PrEP demonstration study. Hypertension (17.9%) was one of the most common chronic nontransmissible comorbidity. We identified depression in 58% of the participants; depression was significantly higher among the HIV-infected (*p*=0.05).

**Table 2.** Baseline Sociodemographic and Clinical Characteristics of Transgender Women by HIV Serostatus—Transcendendo Cohort, Rio de Janeiro, Brazil, 2015–2017

Demographics	Total *N*=322 (%)	HIV-uninfected *N*=148 (%)	HIV-infected *N*=174 (%)	*p*-value
Age^a^				
18–24	68 (21.1)	38 (25.7)	30 (17.2)	0.128
25–35	132 (41.0)	63 (42.6)	69 (39.7)	
36–45	73 (22.7)	29 (19.6)	44 (25.3)	
>45	49 (15.2)	18 (12.2)	31 (17.8)	
Gender identity				<0.001
Woman	74 (23.0)	49 (33.1)	25 (14.4)	
Transgender woman	112 (34.8)	53 (35.8)	59 (33.9)	
Travesti	128 (39.8)	43 (29.1)	85 (48.9)	
Other definitions	8 (2.5)	3 (2.0)	5 (2.9)	
Self-declared race/color				0.111
White	80 (24.8)	39 (26.4)	41 (23.6)	
Black	73 (22.7)	26 (17.6)	47 (27.0)	
Mixed	161 (50.0)	81 (54.7)	80 (46.0)	
Other	8 (2.5)	2 (1.4)	6 (3.4)	
Years of education^a^				0.023
<4	19 (5.9)	5 (3.4)	14 (8.0)	
4–8	100 (31.1)	39 (26.4)	61 (35.1)	
9–12	177 (55.0)	87 (58.8)	90 (51.7)	
>12	26 (8.1)	17 (11.5)	9 (5.2)	
Income (in US$/day)^a,b^				<0.001
>10.00	55 (17.1)	34 (23.0)	21 (12.1)	
1.91–10.00	161 (50.0)	85 (56.1)	78 (44.8)	
≤1.90	39 (12.1)	19 (12.8)	20 (11.5)	
Missing	67 (20.8)	12 (8.1)	55 (31.6)	
Internal migrants^c^	87 (27.0)	45 (30.4)	42 (24.1)	0.256
Age at sexual debut^a^				0.757
<10	226 (70.2)	100 (67.6)	126 (72.4)	
10–13	23 (7.1)	12 (8.1)	11 (6.3)	
14–17	7 (2.2)	4 (2.7)	3 (1.7)	
≥18	66 (20.5)	32 (21.6)	34 (19.5)	
Ever suffered physical violence	174 (54.0)	74 (50.0)	100 (57.5)	0.219
Ever suffered sexual violence (rape)	149 (46.3)	66 (44.6)	83 (47.7)	0.656
Engagement in sex work				0.002
Never	69 (21.4)	44 (29.7)	25 (14.4)	
Current	150 (46.6)	58 (39.2)	92 (52.9)	
Past	103 (32.0)	46 (31.1)	57 (32.8)	
Current syphilis				
No	184 (57.1)	106 (71.6)	78 (44.8)	< 0.001
Yes	88 (27.3)	35 (23.6)	53 (30.5)	
Missing	50 (15.5)	7 (4.7)	43 (24.7)	
Rectal chlamydia				0.004
No	241 (74.8)	121 (81.8)	120 (69.0)	
Yes	40 (12.4)	18 (12.2)	22 (12.6)	
Missing	41 (12.7)	9 (6.1)	32 (18.4)	
Rectal gonorrhea				0.002
No	263 (81.7)	132 (89.2)	131 (75.3)	
Yes	19 (5.9)	8 (5.4)	11 (6.3)	
Missing	40 (12.4)	8 (5.4)	32 (18.4)	
Hepatitis B				< 0.001
No	268 (83.2)	140 (94.6)	128 (73.6)	
Yes	9 (2.8)	1 (0.7)	8 (4.6)	
Missing	45 (14.0)	7 (4.7)	38 (21.8)	
Hepatitis C				0.157
No	307 (95.3)	145 (98.0)	162 (93.1)	
Yes	11 (3.4)	2 (1.4)	9 (5.2)	
Missing	4 (1.2)	1 (0.7)	3 (1.7)	
Diabetes mellitus	7 (2.2)	2 (1.4)	5 (2.9)	0.459
Dyslipidemia	24 (7.5)	8 (5.4)	16 (9.2)	0.281
Systemic arterial hypertension	54 (17.9)	26 (18.3)	28 (17.6)	0.994
Obesity/overweight				
No	170 (52.8)	73 (49.3)	97 (55.7)	
Yes	138 (42.9)	73 (49.3)	65 (37.4)	0.011
Missing	14 (4.3)	2 (1.4)	12 (6.9)	
Depression^d^	186 (57.8)	77 (52.0)	109 (62.6)	0.054
Problematic use of substances (ASSIST score^d^)^e^				
Alcohol	55 (21.5)	18 (16.4)	37 (25.3)	0.115
Tobacco	145 (56.6)	54 (49.1)	91 (62.3)	0.047
Cannabis	74 (28.9)	22 (20.0)	52 (35.6)	0.010
Cocaine	61 (23.8)	15 (13.6)	46 (31.5)	0.002
Amphetamine stimulants	7 (2.7)	2 (1.8)	5 (3.4)	0.702

^a^ Continuous variables were reclassified as categorical.

^b^ US$1.00=R$3.85.

^c^ Participants born outside Rio de Janeiro state were classified as internal migrants.

^d^ Screening by CES-D10.

^e^ ASSIST score of 11+ for alcohol and 4+ for other substances.

ASSIST, Alcohol, Smoking and Substance Involvement Screening Test.

Compared to those HIV-uninfected, HIV-infected trans women had lower schooling and income, reported more engagement in sex work, and had higher rates of STIs. HIV-uninfected and HIV-infected trans women significantly differed in gender identity, as HIV-uninfected trans women more frequently self-reported as women (33.1% vs. 14.4%) and less frequently as travestis (29.1% vs. 48.8%) and had higher obesity/overweight prevalence (Table 2).

A high number of trans women reported problematic use of tobacco (56.6%), cannabis (28.9%), cocaine (23.8%), and alcohol (21.5%) (Table 2). Compared to those HIV-uninfected, higher proportions of HIV-infected trans women had problematic use of cannabis, cocaine, and tobacco.

The majority of trans women perceived themselves as transgender during childhood (63.7% aged ≤10), and the median age of gender transition was 16 years (IQR 14–18) (Table 3). Any feminizing procedure was reported by 41.0%, with only 5.9% reporting SRS. SRS was more frequent among HIV-uninfected than in HIV-infected trans women. About a third had gender-affirming surgery, and 49.1% reported prior industrial silicone filler injection. Most trans women have used hormones at some point, and around half (49.1%) were on current FHT, mostly nonprescribed by health professionals (78.7%). Fewer HIV-infected trans women were currently using hormones (both prescribed and nonprescribed) compared with HIV-uninfected trans women (40.2% vs. 59.5%; *p*<0.001). Among trans women currently using hormones (*N*=158), most of them were using inadequate FHT (57.6% were using intramuscular estradiol plus progestagen, 37.3% reported current use of ethinylestradiol). Only 13.9% reported the use of an adequate regimen (estradiol plus antiandrogen) and 9.5% were under current medical-guided hormonal use. Among trans women currently on hormones, 56.3% and 29.7% achieved the target testosterone and estradiol levels, respectively; 16.5% had estradiol levels higher than recommended.

**Table 3.** Transitioning Characteristics of Transgender Women According to HIV Serostatus—Transcendendo Cohort, Rio de Janeiro, Brazil, 2015–2017

Transitioning characteristics	Total *N*=322	HIV-uninfected *N*=148	HIV-infected *N*=174	*p*
Age of gender perception^a^				
≤7	112 (34.8)	56 (37.8)	56 (32.2)	0.254
8–10	93 (28.9)	39 (26.4)	54 (31.0)	
11–13	66 (20.5)	29 (19.6)	37 (21.3)	
≥14	47 (14.6)	24 (16.2)	23 (13.2)	
Missing	4 (1.2)	0 (0.0)	4 (2.3)	
Age of gender transition^a^				
<14	59 (18.3)	24 (16.2)	35 (20.1)	0.151
14–17	148 (46.0)	63 (42.6)	85 (48.9)	
≥18	114 (35.4)	61 (41.2)	53 (30.5)	
Missing	1 (0.3)	0 (0.0)	1 (0.6)	
Ever performed gender affirmation procedures^b^	132 (41.0)	59 (39.9)	73 (42.0)	0.790
Ever had sex reassignment surgery^b^	19 (5.9)	15 (10.1)	4 (2.3)	0.006
Ever injected fillers	158 (49.1)	57 (38.5)	101 (58.0)	<0.001
Hormone use				
Current	158 (49.1)	88 (59.5)	70 (40.2)	0.002
Past	147 (45.7)	52 (35.1)	95 (54.6)	
Never	16 (5.0)	8 (5.4)	8 (4.6)	
Missing	1 (0.3)	0 (0.0)	1 (0.6)	
Medically guided hormone use				0.014
Never	240 (78.7)	100 (71.4)	140 (84.8)	
Current	29 (9.5)	21 (15.0)	8 (4.8)	
Past	19 (6.2)	10 (7.1)	9 (5.5)	
Missing	17 (5.6)	9 (6.4)	8 (4.8)	
Current use of ethinylestradiol^c^	59 (37.3)	35 (39.8)	24 (34.3)	0.587
Current use of IM estradiol plus progestagen^c^	91 (57.6)	45 (51.1)	46 (65.7)	0.093
Current use of estradiol plus antiandrogen^c^	22 (13.9)	16 (18.2)	6 (8.6)	0.133
Total testosterone^a,c^				
At target level	89 (56.3)	53 (60.2)	36 (51.4)	0.221
Above target level	64 (40.5)	34 (38.6)	30 (42.9)	
Missing	5 (3.2)	1 (1.1)	4 (5.7)	
Estradiol^a,c^				
Below target level	80 (50.6)	47 (53.4)	33 (47.1)	0.433
At target level	47 (29.7)	26 (29.5)	21 (30.0)	
Above target level	26 (16.5)	14 (15.9)	12 (17.1)	
Missing	5 (3.2)	1 (1.1)	4 (5.7)	

^a^ Continuous variables were reclassified as categorical.

^b^ Breast augmentation, facial feminization, tracheal shave, or sex reassignment surgery (vaginoplasty, penectomy, and oorchiectomy).

^c^ Evaluated to trans women currently using hormones (*n*=158).

IM, intramuscular.

Among HIV-infected trans women, median time since HIV diagnosis was 1.8 years (IQR 0–7.3); the median CD4^+^ count was 582 cells/mm^3^ (IQR 372–800); and 60% of them were using ART. Out of the 70 HIV-infected trans women who were not on ART, 38.6% (27) had been diagnosed with HIV on the same day of Transcendendo enrollment, 57.1% (40) had already been diagnosed with HIV but had never used ART, and 4.3% (3) had been on ART before but were not currently using it. Among those currently using ART, 67.3% had undetectable VL (Table 4). ART use was less frequent among younger HIV-infected trans women, but among those who used ART, they were more likely to have undetectable VL: 80.0% among those aged 18–24 versus 57.1% among those aged ≥46. Forty (23.0%) HIV-infected trans women reported a previous AIDS-defining illness; 16.7% reported previous tuberculosis.

**Table 4.** Clinical Characteristics of HIV-Infected Transgender Women, According to Age—Transcendendo Cohort, Rio de Janeiro, Brazil, 2015–2017

Clinical characteristics	Total *N*=174 (%)	18–24 *N*=30 (%)	25–35 *N*=69 (%)	36–45 *N*=44 (%)	46+*N*=31 (%)	*p*
Mode of HIV acquisition						
Sex with men	161 (92.5)	27 (90.0)	66 (95.7)	43 (97.7)	25 (80.6)	0.042
IDU	2 (1.1)	0 (0.0)	0 (0.0)	0 (0.0)	2 (6.5)	
Other/unknown	11 (6.5)	3 (10.0)	3 (4.3)	1 (2.3)	4 (12.9)	
Current CD4 count (cells/mm^3^)^a^						
<250	21 (12.1)	2 (6.7)	11 (15.9)	4 (9.1)	4 (12.9)	0.884
251–500	36 (20.7)	6 (20.0)	15 (21.7)	9 (20.5)	6 (19.4)	
>500	84 (48.3)	16 (53.3)	29 (42.0)	25 (56.8)	14 (45.2)	
Missing	33 (19.0)	6 (20.0)	14 (20.3)	6 (13.6)	7 (22.6)	
Currently on ART						
No	70 (40.2)	25 (83.3)	36 (52.2)	6 (13.6)	3 (9.7)	<0.001
Yes	104 (59.8)	5 (16.7)	33 (47.8)	38 (86.4)	28 (90.3)	
Current HIV RNA viral load (copies/mL) (*N*=103)^b^						
<40	70 (67.3)	4 (80.0)	23 (69.7)	27 (71.1)	16 (57.1)	0.775
≥40	22 (21.2)	1 (20.0)	5 (15.2)	8 (21.1)	8 (28.6)	
Missing	12 (11.5)	0 (0.0)	5 (15.2)	3 (7.9)	4 (14.3)	
Had previous AIDS-defining illness	40 (23.0)	2 (6.7)	8 (11.6)	17 (38.6)	13 (41.9)	<0.001
Had previous TB^c^	29 (16.7)	2 (6.7)	11 (15.9)	11 (25.0)	10 (32.3)	0.051

^a^ Continuous variables were reclassified as categorical.

^b^ Calculated only for those on ART.

^c^ After HIV diagnosis.

ART, antiretroviral therapy; IDU, injection drug use.

## Discussion

Our study presents baseline characteristics of the Transcendendo cohort. Our findings reinforce trans women as a disadvantaged and disenfranchised group, with multiple needs that go beyond health itself. Trans women bear a disproportionate burden of adverse outcomes that are sparingly evaluated, mostly by cross-sectional studies.^2^ Although transgender populations face adverse experiences worldwide, the context of marginalization and social exclusion may be even worse in a low- or middle-income country (LMIC), with poor access to hormone use supervision, gender-related interventions, HIV prevention and care, and support for problematic substance use. Transcendendo is the first Latin-American cohort to evaluate the social, demographic, and clinical aspects of HIV-infected and HIV-uninfected trans women.

### Sociodemographic context of exclusion, discrimination, and violence

Our results reveal that the participants of Transcendendo have low education level and income, experience high physical and sexual violence rates, and frequently engage in sex work. These unfavorable conditions are in accordance with other authors.^32–35^ The social context that trans women face is a combination of diverse related factors such as exclusion, stigma, violence, lack of justice, and poor access to education and employment, which usually exacerbate one another and increase vulnerability to HIV and other diseases.^36^ Although Brazilian laws protect transgender rights, transphobia is ingrained diffusely, and Brazil is the leading country of homicides of transgender people, accounting for almost 40% of all reported murders of transgender people worldwide.^37^

### Mental and physical health

We found that depression was very common in our cohort (58%), comparable to those described using similar screening scales (prevalence between 44% and 49%)^14,38,39^ and more prevalent than observed in the cisgender Brazilian population (13.2–20.2%).^40,41^ This finding is in accordance with data from Canada and the United States, which observed a higher prevalence of depression among trans women compared to cisgender population.^14,42^ The factors associated with gender minority stress, such as victimization, rejection, discrimination, and nonaffirmation of gender identity, seem to be associated with depression and other mental health conditions.^43,44^ We found a lower prevalence of hypertension compared to the data from the general population of Brazil,^45^ which may be due to the lower age of our sample. Additionally, diabetes and dyslipidemia were less frequent in our population, compared with other general population studies in Brazil^46,47^ and with the Canadian trans women cohort.^14^ These contrasts may again be related to the demographic characteristics of the populations, such as age and gender, as well as to the differences in definitions adopted by each study.

### Trans-specific interventions

Most trans women used hormones at some point, almost always nonprescribed by health professionals, at higher levels than described in the United States.^48–51^ We also identified alarming rates of industrial silicon filler injection and lower rates of SRS than described in other countries.^52,53^ These differences might reflect worse access to general and transgender-specific health care. Gender-affirming hormone therapy is the main intervention sought by transgender people, since it allows the development of characteristics compatible with their gender identity.^28^ FHT is considered safe under medical supervision,^54^ but nonprescribed hormones are associated with the uptake of inadequate compounds, improper dosing, and lack of monitoring.^48^ In our cohort, most trans women did not achieve the recommended levels of hormones; more than a third was using ethinylestradiol (37.3%) and some had higher estradiol levels than recommended. Ethinylestradiol is not recommended to feminization due to its high thrombogenic risk.^54^ The combination of intramuscular estradiol plus progestagen, commonly used in our setting, is understudied, but might be associated with cardiovascular disease and breast cancer in postmenopausal women.^55^ In summary, the high rates of inadequate use of hormones in our sample is alarming and points to the unmet needs of this underprivileged group.

### HIV infection

Compared with those HIV-uninfected, HIV-infected trans women had lower educational levels, were involved in more sex work, and reported problematic use of some substances. Forty percent were not on ART, especially the younger population with two-thirds of those on ART having an undetectable VL. These results highlight the need for better retention in care, which will ultimately impact HIV transmission. HIV epidemic among trans women is considered a syndemic condition, which generally develops in the context of social disadvantage and inequality.^4^ It has been suggested that syndemics may also explain the difficulties in the treatment of HIV infection apart from other coexisting social, behavioral, and medical conditions that limit successful and sustained engagement in health care.^12^

### Strengths and limitations

The main strengths of this study are the unique evaluation of an extremely marginalized and vulnerable population, the use of a standardized data collection platform, and the systematic medical and laboratory assessments. Very few studies have evaluated trans women longitudinally, none in LMICs. In addition, trans women are a hard-to-reach and hard-to-retain population for health care.^56–58^ So, to improve retention and outcome assessment, we established a welcoming, gender-affirming setting, which facilitated the engagement and retention of trans women. Also, we established a linkage with the state-level mortality database to improve lost to follow-up evaluation in our population, since it can help identify deaths not reported to our study team. Our study also has some limitations. Foremost, Transcendendo is an open cohort restricted to a convenience sample from the Rio de Janeiro population, and therefore the findings cannot be generalizable to the whole trans women population. Also, as this is a clinic-based cohort, procedures are linked to medical care, which may lead to some missing data. However, to minimize these occurrences, our study procedures are carried out annually.

## Conclusions

Transcendendo is a unique opportunity to longitudinally assess transgender health outcomes among young trans women from a LMIC, including issues regarding hormone and antiretroviral use, incident comorbidities, and complications such as metabolic and cardiovascular events. Baseline results contribute to bridging the gaps concerning transgender people's health and reinforce that transgender care cannot be disentangled from the social environment that surrounds trans women. HIV infection is a major concern among trans women, but transgender members claim that their marginalization needs to be addressed as the highest priority to succeed in a response to HIV.^36^ Transcendendo results may guide future research and trans-specific interventions by addressing transgender vulnerabilities and needs and supporting the development of strategies to diminish stigma, improve health care environment, guide future research on trans morbidities, substance use, feminizing hormone therapy, and trans-specific interventions to support health-related recommendations.

## Abbreviations Used

ART: antiretroviral therapy
ASSIST: Alcohol, Smoking and Substance Involvement Screening Test
FHT: feminizing hormonal therapy
IDU: injection drug use
IM: intramuscular
IQR: interquartile range
LMIC: low- or middle-income country
RDS: respondent-driven sampling
SRS: sex reassignment surgeries
STI: sexually transmitted infection
